# Silencing of Nucleostemin by siRNA Induces Apoptosis in MCF-7 and MDA-MB-468 Cell Lines

**DOI:** 10.22037/ijpr.2020.1100950

**Published:** 2020

**Authors:** Mahdiyeh Moudi, Ramin Saravani, Saman Sargazi

**Affiliations:** a *Genetics of Non-Communicable Disease Research Center, Zahedan University of Medical Sciences, Zahedan, Iran. *; b *Department of Medical Genetic, School of Medicine, Shahid Sadoughi University of Medical Sciences, Yazd, Iran. *; c *Cellular and Molecular Research Center, Resistant Tuberculosis* *Institute, Zahedan University of Medical Sciences, Zahedan, Iran. *; d *Department of Clinical Biochemistry, School of Medicine, Zahedan University of Medical Sciences, Zahedan, Iran.*

**Keywords:** Apoptosis, Breast cancer, Nucleostemin, siRNA

## Abstract

One of the most important modulators involved in controlling apoptosis induction and viability of cancerous cells is nucleostemin (NS). Some studies revealed that NS is also needed to maintain the proliferation of embryonic neural stem cells and early embryogenesis. This study was designed to better elucidate the association between NS depletion status and apoptosis induction of both MCF-7 and MDA-MB-468 cell lines. We examined the effects of NS-targeting siRNAs on the expression of NS in MCF-7 and MDA-MB-468 human breast cancer cell lines by the Real-time polymerase chain reaction method. In addition, we investigated the correlation between knockdown of NS and viability rates and apoptosis induction in MCF-7 and MDA-MB-468 cell lines using the MTT assay and annexin V/PI staining, respectively. The NS-targeting siRNAs inhibited the viability of the cells in a dose- and time-dependent manner and induced apoptosis after 48 h in the cells. Thus, consistent with previous articles, this protein can be one of the regulators related to the inhibition of apoptosis and the increased viability of tumor-initiating cells in human breast cancer cell lines as well as other cancers.

## Introduction

Aggressive breast cancer is the cause of ten thousand of death in women, annually (1). The disease is characterized by an accumulation of abnormal epithelial cells as a result of multistep genetic alterations in the initiating cells. According to the World Health Organization (WHO) classification, there are several criteria for breast cancer classification (2). One of the known criteria is based on estrogen receptor (ER), progesterone receptor (PR), and human epithelial receptor 2 (HER2) statuses. Response to chemotherapy in metastatic breast cancer is controversial since unlike other solid tumors, estrogen receptor positive (ER+) breast cancer patients with skeletal metastasis showed beneficial responses to chemotherapy demonstrating favourable prognosis (3, 4). Unfortunately, this hypothesis was not well established in patients with ER- breast cancer and/or patients with skeletal metastasis (4). 

Two known breast cancer cell lines, which were studied in several papers, are MCF-7 and MDA-MB-468 cell lines. MDA-MB-468 is a basal type triple-negative (ER, PR and HER2 negative) breast cancer cell line while MCF7 is the luminal type expressing estrogen and progesterone receptors (5). MDA-MB-468 cell line has genomic instability because of mutation in BRCA or *TP53 *gene. MCF-7 and MDA-MB-468 cell lines are characterized by uncontrolled proliferation, indefinite self-renewal, impaired differentiation, and inhibits apoptosis. Furthermore, in cancers, one of the stem cell features is indefinite self-renewal. Among the most important modulators involved in controlling the self-renewal of stem cells is NS (*NS*) (6). NS or GTP-binding protein is present in nucleoli, which is thought to regulate stem cell fate via the activation of the p53-dependent pathway (7), although some p53-independent mechanisms have been reported in cancerous cells (8). 

In 2013, a study reported that NS depletion resulted in sphere-forming activity in MCF-7 and MDA-MB-231 cell lines. Several studies showed that senescence and apoptosis, cell cycle arrest, and eventually, embryonic death at the blastocyst stage are affected by the deletion of *NS* in mouse embryonic stem cells (ESCs) (9). Also, some studies showed that knockdown of *NS* expression in cancerous cells could induce apoptosis and cell cycle arrest (10). Therefore, NS appears to play an important role in cell cycle progression in neural stem cells (NSCs) (11), embryonic stem cells (ESCs)(9), and various cancer cell lines (12, 13). Recently, scientists reported the high level of *NS* expression in MCF-7 and breast cancer tissue, suggesting that this nuclear protein might be an attractive molecular target for developing tumor cells. We designed this study to better understand the association between *NS* expression status and apoptosis induction in both MCF-7 and MDA-MB-468 cells.

## Experimental

*Cell line and cell culture*

MCF-7 and MDA-MB-231 cell lines were purchased from the cell repository of the research institute of biotechnology (Ferdowsi University of Mashhad, Iran). Cells were cultured in RPMI1640 medium with 10% fetal bovine serum (FBS) (Rockville, MD, USA), 100 μg/mL streptomycin, 100 U/mL penicillin (Sigma) and maintained at 37 °C in a humidified atmosphere with 5% CO2 until reaching 80% of confluency.

*siRNAs*

The siRNAs targeted human *NS* (Cat.no: SI04319504, SI04298938, SI04211872 and SI03169593, 20 µM) and scrambled negative siRNA (SI03650325, 20 µM) were purchased from Qiagen company (USA). The lyophilized siRNAs were dissolved in RNase free water to a final concentration of 20 µM. siRNA transfection was performed using HiPerFect transfection reagent (Qiagen, Cat No: 301704, USA), as described in the manufacturer’s instructions. In order to find the optimum concentrations of siRNA, a 0.2 µM siRNA stock was prepared from a 20 µM stock. Briefly, different concentrations of 0.2 µM siRNA stock were spotted in each well of 6-wells plates for qPCR and apoptosis assays (Zhejiang Sorfa Medical Plastic Co., Ltd, Zhejiang, China). Then, 12 μL of HiPerFect transfection reagent (Qiagen, USA) was mixed with 100 μL of RPMI1640 without FBS, and the mixture incubated for 15 min at room temperature. The resulting mixture was added dropwise into each well. Finally, 100,000 cells/well in 2300 μL of 10% FBS-contained RPMI1640 medium were added to each well and were allowed to adhere overnight (reverse transfection. The cells were incubated for 6 to72 h after transfection (in 5% CO_2_, 95% humidified air at 37 °C) before additional analyses.

*Real-time quantitative PCR (q-PCR)*

Total RNA was extracted using the RNX plus kit (Cinagen, Tehran). The RNA converted to cDNA using RT-PCR kit from Takara (TaKaRa, Dalian, China). The RNA quality and integrity assessed by NanoDrop (Eppendorf, USA) and Gel electrophoresis. AlleleID software and NCBI-BLAST database were employed in order to design the primers and verify their specificity. The efficiency of primers was validated and a standard curve plotted before running PCR products on 2% agarose gel. RT-qPCR performed using SYBR Green Real-time PCR (Fermentas, USA) by 96-wells ABI machine (Applied Biosystems) under following cycle conditions: 95 °C for 10 min, followed by40 cycles at 58 °C for 30 s and 72 °C for 30 s. The results normalized against *GAPDH* expression. Each qPCR was repeated at least three times for different experimental samples, and each reaction performed in triplicate. We used 2^− (Ct control – Ct treatment) Reference- (Ct control – Ct treatment) target ^method. The sequence for forward and reverse primers of *NS*, *GAPDH,* and *TP53 *are shown in Table 1.

*MTT cytotoxic assay*

Briefly, various concentrations of siRNA solutions (i.e., 25, 50, 100, 200, and 400 nmol/μL) were spotted in each well of 96-well plates for transfection. In a sterile microtube, 1.5 μL of HiPerFect transfection reagent (Qiagen, USA) was mixed with 25 μL of RPMI1640 without FBS. Then, after 15 min of incubation at room temperature for liposome formation, the resulting mixture was added dropwise into each well. Finally, 5,000 cells/well in 175 μL of 10% FBS-contained RPMI1640 medium were added to each well and were allowed to adhere overnight. After 24, 48, and 72 h, 20 μL of MTT (3-(4,5-dimethylthiazol-2-yl)-2,5-diphenyltetrazolium bromide) dye (5 mg/mL) was added and incubated for 3.5 h. Afterward, 200 μL of dimethyl sulfoxide (DMSO) was added for solubilizing the formazan crystals. Following 15 min of incubation at room temperature, the absorbance was measured at 570 nm with a microplate reader. Also, cells treated with irrelevant siRNA were considered as controls.

*Evaluation of the apoptosis*

Apoptosis assays performed using annexin V- PI apoptosis Assay Kit (Thermo Fisher, USA). For this purpose, the number of viable and non-viable cells for each well was counted using the trypan blue test; then, 2 × 10^4^ cells were seeded in each well of 6-well plates. After 48 h of treatment of cells with *NS*-siRNA (*NS*-targeting small interfering RNA) and irrelevant siRNA (IR-siRNA), the cells were washed in cold phosphate-buffered saline (PBS), and then gently trypsin solution (1%) was added to control transfected cells. Then, the trypsinized cells were collected and re-suspended in 200 μL of binding buffer and 5 μL of fluorochrome-conjugated annexin V (1 μg/mL). The mixture was incubated at room temperature for 15 min in the dark, and then 5 μL of PI (Propidium Iodide) was added. The cells were analyzed using a flow cytometer within 30 min of staining.

*Statistical analysis*

Statistical analysis was performed with SPSS version 16. An independent sample t-test and the Friedman test were used for comparison of mean ± SD values. The statistically significant level was considered* P<0.05*. All experiment was done at least in triplicates.

## Results

*Silencing of NS in breast cancer cells*

**Figure 1 (A):**
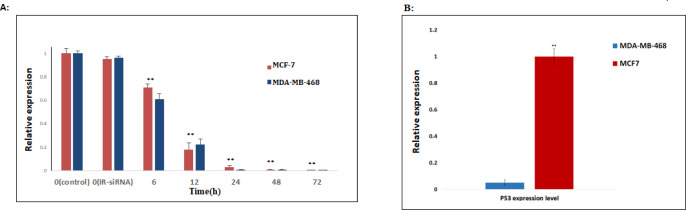
*NS*-siRNA reduced *NS* expression in MCF-7 and MDA-MB-468 human breast cancer cells. (A) *NS* expression levels in MCF-7 and MDA-MB-468 cells transfected by *NS*-siRNAs were assessed by real-time quantitative PCR at 6, 12, 24, 48, and 72 h after transfection. Also, the expression level of *NS* was assessed in the cells transfected by irrelevant-siRNA (IR-siRNA) and not transfected (control). The results were normalized to *GAPDH* and presented as the mean ± SD of three independent experiments. (B): The expression level of *TP53 *in MDA-MB-468 cells significantly was down-regulated compared to MCF-7 cells. ** *p-*valu*e *< 0.05 compared to the untreated cells and cells treated with irrelevant (IR)-siRNA

First, we checked the effects of the *NS*-siRNAs on the expression of endogenous *NS* in MCF-7 human breast cancer cell lines, which had been transfected with 80 nmol/μL of *NS*-siRNA and the control dsRNA. At 6, 12, 24, 48, and 72 h after transfection, the expression of *NS* mRNA was assessed by real-time quantitative PCR. Compared with IR-siRNA, *NS*-siRNAs first caused a significant reduction in the expression level of the *NS* at 12 h after transfection (0.22 ± 0.05, *p-Value*<0.001) and following at 72 h after transfection, the knockdown of *NS* was observed. Also, the application of *NS*-siRNAs resulted in a significant reduction in *NS* expression in MDA-MB-468 cells compared to the cells treated with IR-siRNA and control (0.17 ± 0.06, *p-value *= 0.03) (Figure 1A). The optimal knockdown of *NS* in both MCF-7 and MDA-MB-468 cells was seen 72 h post-transfection (0.0001 ± 0.008, 0.0009 ± 0.009, respectively).

*Decreased expression of TP53 mRNA in MDA-MB-468 compared to MCF-7 cells*

To compare the expression of *TP53 *between the MCF-7 and MDA-MB-468 cells, mRNA levels of the *TP53 *was assessed in both MCF-7. Real-time PCR analysis indicated that MCF-7 cells express a comparable level of *TP53 *related to MDA-MB-468 cells, although the apoptosis induction of MDA-MB-468 cells is significantly less efficient than that of MCF-7 cells. (0.043 ± 0.03, *p-Value<0.05*). (Figure 1B)

*Knockdown of NS inhibits MCF-7 and MDA-MB-468 cells proliferation*

**Figure 2:**
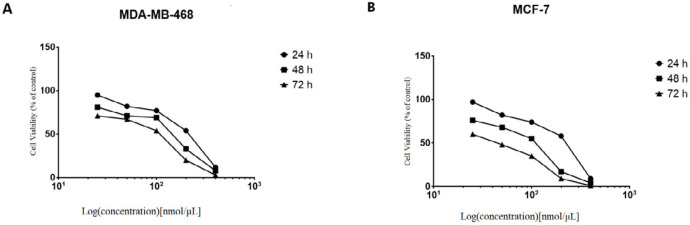
NS-siRNAs inhibited the viability of cells in a dose-dependent and time-dependent manner, which assessed by the MTT assay in **(A)** MCF-7 and **(B)** MDA-MB-468 cells. The percentage of living cells was reduced after treatment with *NS*-siRNAs (25 to 400 nmol/µL) at 24, 48, and 72 h. The data are presented as means ± SD

We next investigated the effects of knockdown of *NS* on the breast cancer cells. The four siRNAs targeting *NS *mRNA and IR-siRNA were respectively transfected into the MCF-7 cells at the concentrations of 25-50-100-200-400 nmol/μL. The effects of the *NS*-siRNAs at varying concentrations and times (24 to 72 h) on the proliferation and viability of MCF-7 and MDA-MB-468 cells were determined via MTT assay. Inhibition of cell growth and viability by *NS*-siRNAs was concentration and time-dependent (Figure 2). Reduction in MCF-7 cell viability via *NS*-siRNA treatment at increasing concentrations following 48 h ranged from 3 % to 79 %, whereas reduction of cell viability in MDA-MB-468 cells ranged from 7% to 88 % (Figure 2). Increasing concentrations resulted in a decrease in the percentage of viable cells. A similar cytotoxicity trend was also observed when the cells were treated with *NS*-siRNAs. The MCF-7 cells were more sensitive toward knockdown of *NS* than MDA-MD-468 cells (Figure 2). The IC50 values for the mixture of *NS*-siRNAs were 79.93 and 118.4 nmol/μL on MCF-7 and MDA-MB-468 cells, respectively.

*Silencing of NS induces cell apoptosis in breast cancer cells*

**Figure 3:**
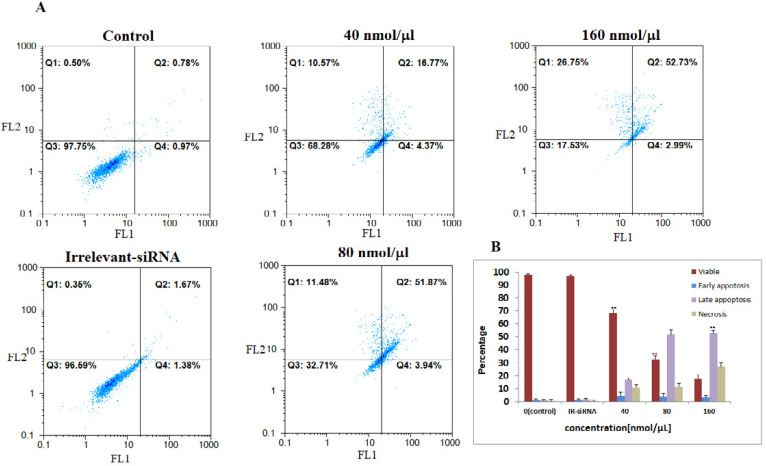
The four *NS*-siRNAs induced apoptosis in MCF-7 cells. A: *NS*-siRNAs treatment resulted in the significant apoptosis in MCF-7 cells at 40, 80, and 160 nmol/μL concentrations detected using a double-staining method with fluorescein thiocyanate-conjugated annexin V and propidium iodide. The percentage of the living cells transfected by irrelevant-siRNA was 95.69%. Also, 97.75% of non-transfected (control) cells were viable. B: A statistical graph of annexin V-FITC/PI staining in MCF-7 is shown. The data averages for each time point were calculated using the results from three independent experiments. The results are expressed as the mean ± SD. Apoptotic cells included the Annexin V+/PI− cells and the Annexin V+/PI+ cells. **: *p*-Value<0.05 compared to the control group

**Figure 4:**
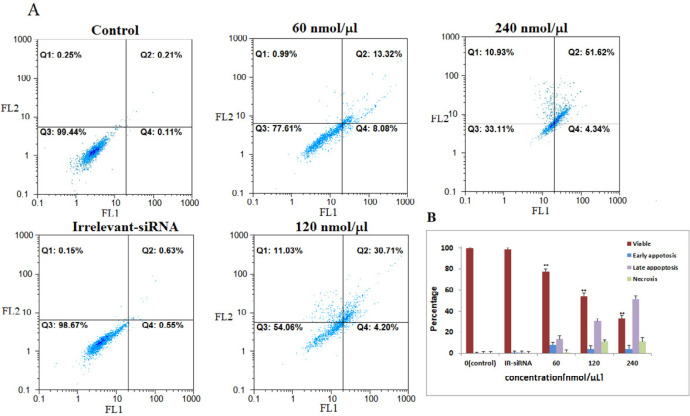
The four siRNAs targeting *NS *mRNA induced apoptosis in MDA-MB-468 cells**. **A: *NS*-siRNAs treatment induced apoptosis in MDA-MB-468 cells at 60-120-240 nmol/µL concentrations detected via annexin V/PI staining method. B: A statistical graph of flow-cytometry results in MDA-MB-468 cells is shown. These data are the average results of three independent experiments. The results are expressed as the mean ± SD. The data are presented as the mean ± SD. **: *p-*Value<0.05 compared to the control group

Then, we assessed the relationship between *NS*-siRNAs-mediated loss of apoptosis by annexin V/PI staining flow-cytometry analysis of MCF-7 and MDA-MB-468 cells labeled with PI and FITC. The *NS*-siRNAs and IR-siRNA were transfected into MCF-7 cells at 40, 80, and 160 nmol/μL concentrations and into MDA-MB-468 at 80, 160, and 240 nmol/μL concentrations. We found that the cells treated with concentrations higher than 80 nmol/μL of NS-siRNAs underwent significant apoptosis following 48 h treatment in both MCF-7 and MDA-MB-468 cells (*p-*value < 0.05). The number of early apoptotic cells increased to 4.37% in MCF-7 cells and 8.08% in MDA-MB-468 and the number of late apoptotic cells increased to 16.77% in MCF-7 cells and 13.02 % in MDA-MB-468. These data also showed that 52.73% of MCF-7 cells treated with *NS*-siRNAs and 51.62% of MDA-MB-468 cells treated with *NS*-siRNAs at 160 and 240 nmol/μL concentrations after 48 h were of late apoptotic cells (Figure 3, 4).

**Table 1:** Primer sequences of *TP53*, *NS* and *GAPDH*

Genes	Sequence	Product length (bp)
***TP53 *** **(Forward)**	CAGCACATGACGGAGGTTGT	67
***TP53 *** **(Reverse)**	CCAGACCATCGCTATCTGAGC	
***NS*** ** (Reverse)**	AAAGCCATTCGGGTTGGAGT	200
***NS*** ** (Forward)**	ACCACAGCAGTTTGGCAGCAC	
***GAPDH*** ** (Forward)**	CATGTAGTTGAGGTCAATGAAGG	150
***GAPDH*** ** (Reverse)**	GAGCCACATCGCTCAGACAC	

## Discussion

This study revealed that *NS* depletion inhibits cell viability in MCF-7 and MDA-MB-468 human breast cancer cell lines at the concentrations of 25-400 nmol/μL; the decline in the viability of MCF-7 and MDA-MB-468 cell lines after knockdown of *NS* was entirely consistent with the literature. For instance, cell viability and cell growth decreased after *NS* knockdown in cervical cancer, bladder cancer, prostate cancer, and leukemia as well as many stem cells, such as human ESCs and hematopoietic stem cells (8, 10, 12, 13). Thus, these findings suggest that *NS* plays a critical regulatory role in the cell viability of cancers, especially MCF-7 and MDA-MB-468 cell lines.

Concerning previous studies, *NS* expression had increased in the MCF-7 cell line upon 17β Estradiol treatment and human breast cancer tissue (14). *NS* encodes a nucleolar GTP-binding protein abundantly expressed by cancers and stem cells, identified as a gene enriched in NSCs (11). Some studies revealed that NS is also needed for maintaining the proliferation of embryonic NSCs and human cancer cells and early embryogenesis (7). Therefore, the increased *NS* expression seems to be up-regulated in stem-like cells in tumor cells, better known as cancer stem cells or tumor-initiating cells (15). Also, our data determined that *NS* knockdown induces a late apoptotic response in MCF-7 and MDA-MB-468 cells. Consistent with our data, *NS* depletion after 48 and 72 h of *NS*-siRNAs transfection in K562 cells resulted in delayed apoptotic response (16). In contrast, early apoptosis response was also reported in PC-3 cells and HL-60 cells after knockdown of the *NS* gene (17). These discrepancies could be explained by differences in the knockdown levels of *NS* in different cell lines (higher than 80% in HL-60 and PC-3 cells) and differences in phenotype and proteomics of the cells.

In 2010, Tsai *et al.* reported that knockdown of *NS* reduced the sphere-forming activity of MDA-MB-231 and MCF-7 cells, and *NS* expression was associated with the basal subtype of mammary tumor cells. They also described that tumorigenic activities strongly increased in mammary tumor cells with higher expression levels of *NS* both *in-vitro* and *in-vivo* (15). Also, Antony *et al*, stated that knockdown of *NS* induced higher rates of apoptosis in Her2-transfected MCF-10A cells. Also, western blot analysis of their study indicated that MCF-7 cells express approximately 30% of the levels of *NS *compared to either Her2-transfected MCF-10A or SKBR3 cells. Therefore, it seems to have an essential role in the induction of apoptosis in human breast cell lines, which is consistent with our findings (18).

NS has been recently reported as a novel p53-binding protein (19) and is expressed in invasive breast cancer (20). Some investigations stated that NS is regarded as one of several nuclear proteins that can bind to MDM*2* and therefore stabilize p53 and knockdown of *NS* induces cell cycle arrest/apoptosis in human cancer cells by up-regulation of p53 (19, 21). Some studies reported that *NS* depletion induced p53-independent apoptosis pathways in cancers. For example, Nikpour *et al.* demonstrated that *NS* depletion by siRNA induced a severe decline in cell proliferation and apoptosis in SW1710 cell line with mutated *TP53 *gene (22). Previous investigations revealed that the MDA-MB-468 cell line harbors a bi-allelic mutation in *TP53*, which did not affect its binding affinity to *BRCA1* (23). In another paper, it is reported that the inhibition of *TP53 *mutation leads to apoptosis induction. Thus *TP53 *mutation is required for the survival of MDA-MB-468 cells (24). However, the MCF7 cells are *TP53*-proficient. Also, it is estimated that one-third of breast cancer cases are associated with *TP53 *mutations (25). The genomic instability that was observed in MDA-MB-468 cells mainly activates *ATM* (Ataxia Telangiectasia Mutated) and related proteins. *ATM* activation with the stabilization of p53 and *BRCA1* results in up-regulation of the mutated p53 and *BRCA1* in these cells (26, 27). In this paper, we evaluated the possible correlation between siRNA-induced *NS* depletion and the expression level of *TP53 *in MCF-7 cells (without mutated *TP53*) and MDA-MB-468 cells (harboring mutated *TP53*). The *TP53 *mRNA levels were assessed in MCF-7 compared to MDA-MB-468 cell lines, showing that although *TP53 *expression was significantly decreased in the MDA-MB-468 cells compared to MCF-7 cells prior to treatment with a mixture of NS-siRNAs, knockdown of *NS* induced apoptosis in both cell lines. Thus, in concordance with Nikpour *et al.* findings, our data demonstrated that NS may activate *p53*-independent apoptotic pathways in MDA-MB-468 cell lines lacking *TP53 *expression. Yet, the molecular mechanism of apoptosis induction via *NS* silencing and the link between NS and *TP53 *status in human breast cancer cells remained ill-defined. Therefore, the identification of these associations requires simultaneous *TP53 *silencing and knockdown of *NS* in both MCF-7 and MDA-MB-468 cells.

## Conclusion

In overall, our results suggest a strong correlation between the *NS* signalling pathway and cancer cell survival. These findings provide evidence that knockdown of *NS* can induce apoptosis in MCF7 and MDA-MB-468 breast tumor cells. Thus, in line with original reports, NS may have a novel functional role in cell cycle regulation.
